# Molecular identification and investigations of contagious ecthyma (Orf virus) in small ruminants, North west Ethiopia

**DOI:** 10.1186/s12917-018-1339-x

**Published:** 2018-01-15

**Authors:** Mebrahtu Tedla, Nega Berhan, Wassie Molla, Wudu Temesgen, Sefinew Alemu

**Affiliations:** 1 Department of Biomedical Sciences, University of Gondar, College of Veterinary Medicine and Animal Sciences, P.O. Box: 196, Gondar, Ethiopia; 2 Department of Biotechnology, University of Gondar, College of Natural and Computational Sciences, P.O. Box: 196, Gondar, Ethiopia; 3 Department of Clinical Studies, University of Gondar, College of Veterinary Medicine and Animal Sciences, P.O. Box: 196, Gondar, Ethiopia; 4 Department of Veterinary Epidemiology and Public Health, University of Gondar, College of Veterinary Medicine and Animal Sciences, P.O. Box: 196, Gondar, Ethiopia

**Keywords:** Orf virus, Incidence, Cytopathic effect, PCR, Sheep and goat, Risk factors

## Abstract

**Background:**

Orf virus, the prototype of parapoxvirus, is the main causative agent of contagious ecthyma. Little is known about the status of the disease in Ethiopia and this study was aimed at determining its status using PCR as a confirmatory tool.

**Methods:**

a total of 400 randomly selected sheep and goat was screened for the identification of the virus using amplification of B2L gene and transfection of mammalian cells (VERO cells).

**Results:**

Out of 400 animals screened for infection of the virus, 48 animals were found positive to PCR and revealed an overall incidence of 12%. Different epidemiological parameters were considered to look at the association with incidence of the disease and of which, only species of the animal(sheep), non-vaccinated and non-treated animals, nursing animals, poor body condition animals, extensively managed animals, animals having mouth lesion, and study areas having outbreak history showed higher prevalence. A univariate logistic regression analysis showed statistically significant difference in all variables (*P* < 0.05). Whereas, age and sex of animals showed no significant difference (*P* < 0.05).

**Conclusion:**

The result of the present finding showed high incidence of Orf virus in the region as confirmed through PCR.

## Background

Small ruminants have a tremendous importance in terms of generating income in the form of meat, wool, hide and occasionally milk in small scale farming. However, infectious diseases are still the health challenges by hindering the production and productivity and this causes huge economic loss [1, 2]. Contagious ecthyma or alternatively called contagious pustular dermatitis is a viral disease of sheep and goat caused by Orf virus which belongs to the family of poxviridae and based on the classification of international committee on taxonomy of viruses(ICTV), it is one of the notifiable viral disease known to have a zoonotic importance [1, 3, 4]. It is characterized to have a benign nature, and it can cause a large tumor like vascularized lesions which can be treated using antiviral drugs or removed surgically. It is transmitted through direct contact with infected animals and environmental contamination [5, 6].

Although the rate of morbidity is generally higher than the mortality rate, younger animals such as lambs are more susceptible for the disease and the mortality is significantly higher [7]. Currently, there are live Orf vaccines which can control the disease, but it has a spreading potential to the environment and increasing the risk of another animals [7]. Infected animals can show a common clinical picture such as infectious pustules around the lip area, mouth, tongue, and very rarely on udder and teats and unsurprisingly, the disease in various outbreaks is named as Orf, contagious ecthyma, pustular dermatitis, infectious labial dermatitis, scabby mouth, or sore mouth [8].

The pathogenesis starts with the development of skin erythema and progresses in to vesicle and pustules formation and finally scab formation. One of the typical characteristics of the disease is proliferative and sometimes self-limited lesions on the infected regions of the skin, mucosa of oral cavity and nostrils [9]. The morphology of the virus under electron microscope looks like an ovoid with diagonally woven structures or bands [10, 11].

The virus genome consists of linear double-stranded DNA ranging from 134 up to 139 kb [6]. The envelope gene (B2L) of the virus encodes for a highly immunogenic envelope protein [12]. And amplification of this gene has been used for the detection of ORFV by PCR [13–15]. A gene called vascular endothelial growth factor is identified as a potential cause of Orf virus pathology due to its ability of stimulating endothelial cells to promote vascular permeability [16–18]. A sequencing analysis of the virus has been studied and Peralta et al. 2015 [19] identified five genes namely, B2L, ORFO20, ORF109, ORF127, ORF117 genes from virus outbreaks in Argentina. Furthermore, Delhon G.et al. 2004 [20] sequenced the whole genome of the virus and phylogenic analysis has been carried out from various isolates from around the world such as Korea [21], Brazil [22], china [23, 24] and India [25].

The disease has a global distribution [25–27] and it affects not only sheep and goat but also a wide range of animals such as dog, cattle, camel and wild animals and it is endemic in countries where these animals are common [6]. Outbreaks of contagious ecthyma has been frequently occurred in Ethiopia. However, there is no detail report on the molecular identification of the disease so far and this study was aimed for the first time to identify the status of the disease in the study area.

## Methods

### Identification of study area

**Fig. 1:**
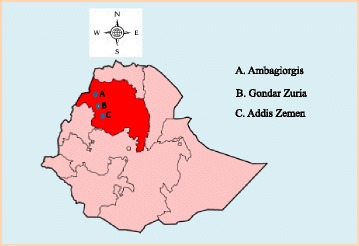
Geographic location of the study areas

The present study was conducted in three districts. Namely, Ambagiorgis from highland, Gondar Zuria from midland, and Addis Zemen from lowland areas. The study areas are depicted in the map showed in Fig. 1, modified from (https://no.wikipedia.org/wiki/Amhara_(region)(region)). The study areas were represented considering presence of adequate study animals and diversity in agroecology.

### Screening and sampling procedure

**Fig. 2:**
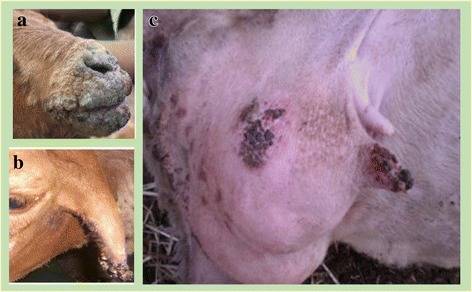
Typical clinical lesions of contagious ecthyma in different body parts. **a**) nostrils and mouth region, **b**) ear, **c**) teat and udder

This study involved a total of 400 randomly selected animals (200 sheep and 200 goat). The sampling procedure involves first step of screening infected animals having a characteristic Orf lesions as shown in Fig. 2 followed by taking tissue samples from the lesion. Fresh lesion scraping was taken immediately from the animal aseptically using diethyl ether. Dried scabs, papular fluids, crusts, and nodules collected were stored in ice box using 1% PBS supplied with pen strep and transported to national veterinary institute for confirmation. Data related to different risk factors or variables were collected through direct observation or by direct interviewing of animal owners. Ethical approval for animal studies was approved by the animal ethics and welfare committee of the University of Gondar. Informed consent was obtained from animal owners who participated in the study.

### Viral isolation, culture and PCR amplification

Tissue samples were first washed using 1% PBS supplied with antibiotics three times and from the third wash, tissues were centrifuged at 14,000 rpm for 10 min. Then 500 μl of viral supernatant was taken for the infection of VERO cells. Cells were cultured on 25cm^2^ culture flask using sterilized GMEM (Sigma Aldrich) and with the addition of 10% FCS. Then cells were incubated for 5 days at 37 °C, 5% CO_2._ The confluency of the cells and the cytopathic effects was regularly checked [28]. DNA was extracted following Commercial Purlink ™ genomic DNA kit(Invitrogen) and purified DNA was stored at -20 °C until the next use.

### Polymerase chain reaction

After isolating genomic DNA from the virus, B2L gene was amplified using forward primers (5′-TGA GCT GGT TGG CGC TGT CCT-3′) and reverse primers (5’-CGC AGA CGT GGC TCA GTA CGT-3′). The reaction set up was prepared as follows: 5X standard reaction buffer (5 μl), 2 mM dNTPs (0.5 μl), 500 nM forward primer (1.25 μl), 500 nM reverse primer (1.25 μl), template DNA (5 μl), 2.5 U Taq DNA Polymerase (0.25 μl), nuclease-free water (to 25 μl). The thermal profile was set as follows: Initial denaturation (94 °C, 5 min, 1X cycle), Denaturation (94 °C, 1 min), Annealing (55 °C, 60 s, 35X), Extension (68 °C, 70 s), Final extension (68 °C, 5 min, 1X).

The size of DNA fragments was estimated using agarose gel electrophoresis. The DNA samples was mixed thoroughly with a loading dye at a ratio of 1:6. The mixed DNA samples were carefully loaded in to the gel and 1 kb plus DNA marker was used to gauge the size of the DNA. The fragments were separated at a voltage of 100 V for a run time of 60 min. DNA bands were analyzed using UV transilluminator.

### Data management and analysis

All date collected from the study were recorded in a microsoft excel spreadsheet and the statistical analysis was performed using STATA version 13. prevalence was calculated by dividing the number of positive animals to total animals examined multiplied by 100. Univariate logistic regression analysis was used to indicate the association of different risk factors with the occurrence of Orf virus infection.

## Results

**Fig. 3:**
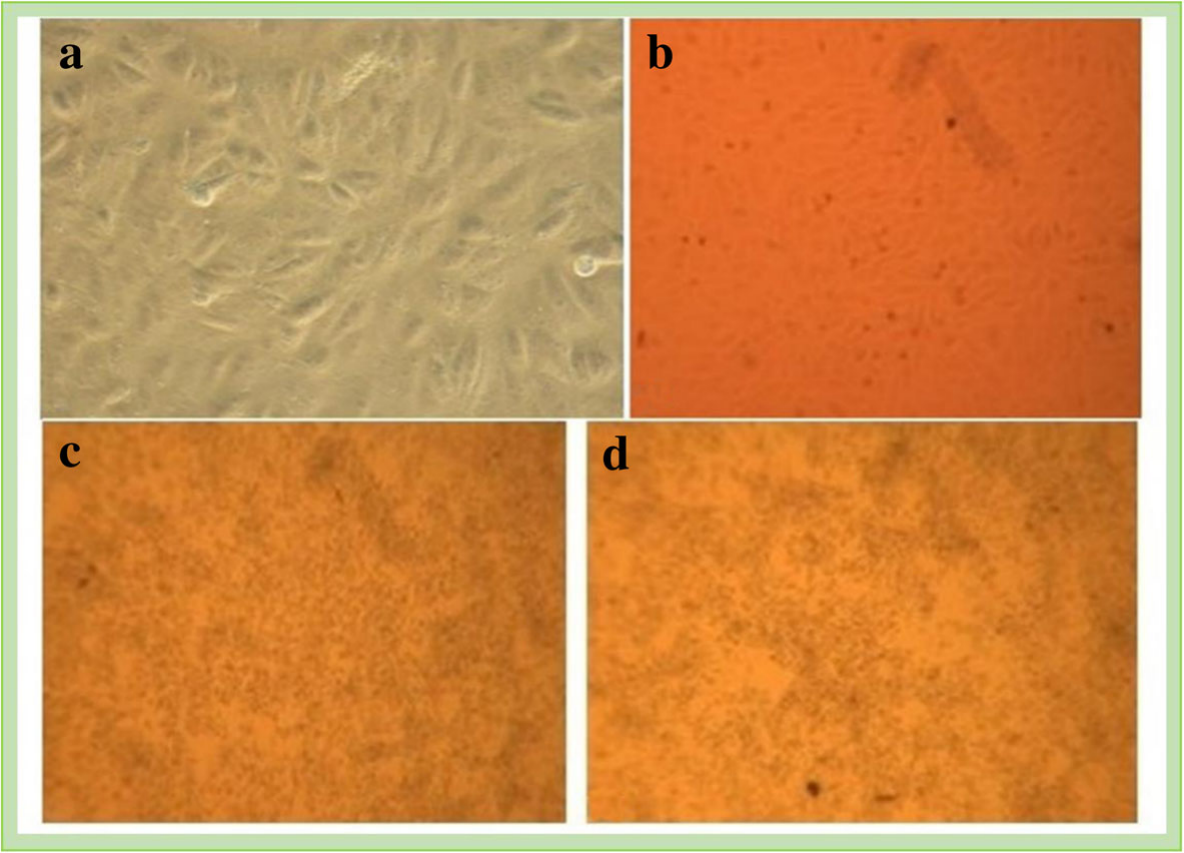
**a**) Normal cells before viral transfection; **b**) Cells are trypsinized and appeared as a single cell; **c** & **d**) showing the peculiar feature of the cytopathic effects after 2 days of incubation

**Fig. 4:**
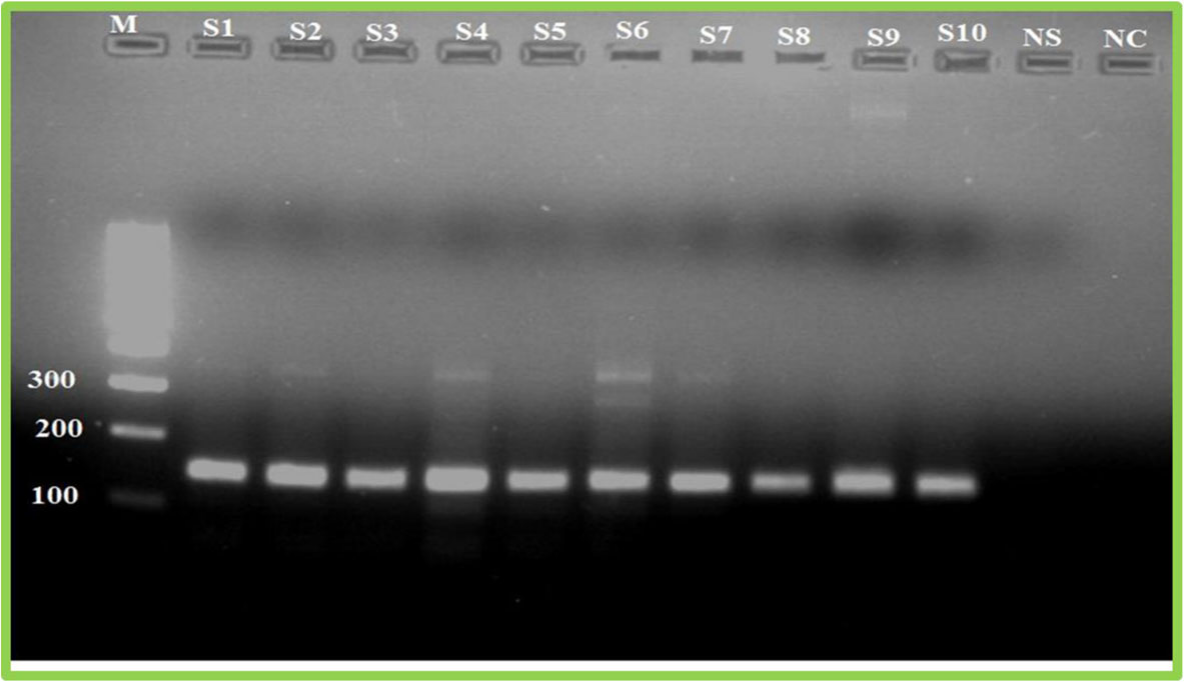
The band size of 140 bp is the amplified product of B2L gene which is a confirmatory for the presence of the Orf virus on the clinical samples. M represents a 10,000 bp molecular weight marker and S1-S10 represents the unknown samples tested and NS is the negative sample. Whereas, NC is the negative control

Cytopathic effect (CPE), characterized by the degenerative changes in VERO cells due to multiplication of the viruses in the cells was used as a first step screening of the samples by examining the morphology of cells after virus transfection of cells and a peculiar feature of evident syncytia formation was observed. Hence, different passages of cells were examined to identify the cytopathic effect of the virus and a total of 48 samples were processed for viral isolation and observation of CPE and the result revealed all the viral suspensions were toxic to the cells which was pre- confirmatory test for the virus. The morphology of VERO cells before and after transfection by the virus is illustrated in Fig. 3. Furthermore, genomic DNA from all clinical samples was extracted to amplify the B2L gene of the virus using a specific primer and revealed a PCR product of 140 bp size as confirmed by agarose gel electrophoresis. A representative sample assigned from S1 to S10 showed samples which were positive for the virus as indicated in Fig. 4.

For investigating the association of the disease with different putative risk factors, a total of 400 animals were screened for the identification of any signs of Orf such as contagious pustular dermatitis in different body parts of the animal including the mouse, nostrils, udder, teat, reproductive organs, interdigital space and the result showed an overall incidence of 12%. In addition, the questionnaire survey administered to the animal owners also indicated that animals having a Orf lesion showed a mortality rate of 4.8%. Orf identification based on the location of the lesion on the animal body revealed a prevalence of 19.8, 10.5, 8.97 and 2% on mouth, nostrils, teat and udder, and lower legs respectively. The univariate logistic regression analysis showed a strong association (*P* < 0.05) between the prevalence of Orf and the lesion development in various organs of the animal body with higher prevalence in mouth and lower prevalence in lower legs of the animal (Table 1).

**Table 1:** Univariate logistic regression analysis of the prevalence of Orf Virus infection with different putative risk factors

Body part lesion	N	No. Positive	Prevalence, % (95% CI)	*P*- value
Mouth	121	24	19.80 (14.71–23.22)	
Nostrils	153	16	10.50 (8.03–12.45)	0.041
Teat and udder	78	7	8.97 (5.98–11.32)	
Lower leg	50	1	2.00 (0.98–3.45)	
Total	400	48	12.00 (11.21–14.87)	
Species				
Goat	200	17	8.5 (7.23–10.11)	0.054
Sheep	200	31	15.5 (13.43–17.26)	
Total	400	48	12.00 (11.21–14.87)	
Sex				
Male	190	21	11.05 (9.86–14.36)	0.32
Female	210	27	12.85 (10.03–15.88)	
Total	400	48	12.00 (11.21–14.87)	
Body condition				
Poor	155	27	17.4 (14.01–19.23)	0.043
Medium	130	12	9.20 (7.01–12.87)	
Good	115	9	7.80 (5.44–9.06)	
Total	400	48	12.00 (11.21–14.87)	
Age				
< 1 year	47	3	6.4 (4.49–9.67)	
1–2 year	133	22	16.5 (12.54–19.98)	0.65
2–3 year	152	14	9.2 (6.31–12.03)	
> 4 years	68	9	13.2 (12.79–15.02)	
Total	400	48	12.00 (11.21–14.87)	
Nursing				
Yes	88	15	17.04 (14.01–21.39)	0.047
No	312	33	10.57 (7.75–11.64)	
Total	400	48	12.00 (11.21–14.87)	
Treatment and Vaccination				
Treated with anthelmentics	121	11	9.1 (7.01–12.20)	0.021
Not treated with anthelmentics	49	7	14.3 (11.10–17.09)	
Treated with antibiotics	112	9	8.0 (6.93–10.05)	
Not treated with antibiotics	35	6	17.14 (13.67–21.39)	
Any vaccination history	46	7	15.21 (12.32–17.66)	
No any vaccination history	37	8	21.62 (17.31–24.89)	
Total	400	48	12.00 (11.21–14.87)	
Management System				
Extensive	106	15	14.15 (11.05–17.08)	0.042
Semi extensive	94	11	11.70 (7.88–13.09)	
Mixed	81	9	11.11 (7.64–13.04)	
Free housing	56	10	18.18 (15.03–21.80)	
Protected housing	63	3	4.76 (2.44–7.42)	
Total	400	48	12.00 (11.21–14.87)	
Study Area				
Gondar Zuria	160	21	13.12 (10.19–15.32)	0.65
Ambagiorgis	135	14	10.37 (7.83–13.09)	
Addis Zemen	105	13	12.38 (8.21–14.39)	
Total	400	48	12.00 (11.21–14.87)	
Type of Treatment		No. Responding		
Moist dressing		32	15.2 (12.1–19.64)	0.032
Surgical removal		57	27.1 (21.3–31.24)	
Culling		26	12.4 (9.02–15.62)	
Antibiotics		95	45.2 (39.56–45.42)	
Total		210		
Outbreak history				
Yes		280	70 (64.10–74.50)	0.012
No		120	30 (27.21–34.52)	
Total		400		

Analysis on the prevalence of Orf virus based on the species of the animal showed a higher prevalence (15.5%) on sheep than goat (8.5%). The statistical association showed species has an impact on the prevalence of the disease (Table 1). Out of 190 male animals examined, 21 (11.05%) were positive for Orf virus and out of 210 female animals examined, 27 (12.85) were positive for the virus (Table 1). Although there was a difference in the prevalence of the virus among both sex groups, no statistically significant association was observed (*P* < 0.05). A higher prevalence (17.4%) of Orf virus was recorded in poor body conditioned animals than medium (9.2%) and good (7.8%) body conditioned animals (Table 1). The statistical analysis of these variables related to the prevalence of the disease showed a significant difference (*P* < 0.05). The prevalence based on age of the animal was recorded, and highest prevalence rate was recorded in the age group of 1–2 years (16.5%) than < 1 years (6.4%), 2-3 years (9.2%), and > 4 years (13.2%). Though there was a slight difference in the prevalence of each age groups, the statistical association showed no significant (*P* > 0.05). The result of the present study showed a prevalence of 17.04% in animals which are nursing. Whereas, a prevalence of 10.57% was recorded in non-nursing animals. The statistical analysis revealed a strong association between the nursing status and prevalence of Orf in the study area (*P* < 0.05). The prevalence of Orf virus in animals having either treatment and vaccination history was studied and showed a prevalence of 9.1% (anthelmintic treated animals), 14. %(no anthelmintic treated animals), 8% (antibiotic treated animals), 17.14%(no antibiotic treated animals), 15.21% (vaccinated animals), 21.62% (non-vaccinated animals). The statistical analysis revealed a significance difference among each group in relation to prevalence of the disease (*P* < 0.05).

By considering the management system of the study animals, we investigated whether there is an effect on the prevalence of the disease or not and the result showed a high prevalence of Orf virus in free housing animals and extensively managed animals with a prevalence of 18.18 and 14.15% respectively. The result of statistical analysis also showed that there was a strong association between management system and prevalence of the disease (Table 1. The prevalence on different study areas also showed a variation in the status of the disease in which higher prevalence was recorded in Addis Zemen (12.38%), Amba Giorgis (10.37%), and Gondar Zuria (13. 12%).However, we statistically found no significant difference in the prevalence of the disease in all the study areas (*P* > 0.05).

Furthermore, a total of 210 animal owners were interviewed on the treatment practices of their animals after they got infected and most (45.2%) use antibiotics followed by surgical removal (27.1%), moist dressing (15.2%) and culling (12.4%) as treatment options. In addition, the response of the owners and animal health workers on the outbreak history of the disease in the area showed a higher prevalence of Orf virus (70%) in areas having an outbreak history and lower (30%) in areas having no outbreak history (Table 1).

## Discussion

In this study, we presented the first clinical report of Orf virus in small ruminants based on molecular identification in north western regions of Ethiopia. The disease has a high morbidity rate but low mortality rate and as a result, less attention has been given by the owners. However, the loss of production and productivity of the animals due to the disease significantly jeopardize the market value. Over the study period, we have recognized lack of adequate diagnosis and effective treatment of clinical cases which is the main contributing factor for the occurrence of disease outbreaks at any time points. The traditional methods of diagnosis which depends on the characteristic clinical signs is inaccurate, but virus isolation and culturing is thought to be a gold standard method of confirmation though it is time-consuming [29]. With the development of molecular biology, PCR technique has become widely used to amplify the desired genomic fragments from tissue specimens, and it has become a powerful tool in molecular diagnosis. This method is recommended for proper identification of the pathogen through gene amplification using specific forward and reverse primers [30]. Various researchers in different parts of the world have reported contagious ecthyma as common outbreak of sheep and goats [31] and indicated that PCR is a quick confirmatory test for Orf virus [32].

In the present study, we showed the distribution of the disease using tests such as the cytopathic effect(CPE) of the virus in mammalian cells followed PCR amplifications of B2L gene. In addition, this study showed the association of different variables that we classified them as risk factors with the incidence and prevalence of the disease. In this study, we found that the infection of Orf virus is common in mouth and nostrils of the animal. This is due to the close confinement and grazing habit of the animals which causes the formation of minor abrasions on the mouth and lips of the animal during feeding. A very low incidence of the virus was also observed on the teat and udder of the animal particularly in nursing animals in which the infected lambs could possibly be the source of infection during suckling. Furthermore, species of the animal showed a significant impact on the prevalence of the disease in which sheep were more sensitive than goat with a prevalence of 15.5% in sheep and 8.5% in goat. Though the prevalence is higher in sheep than goat, the severity of infection is vice versa and this finding is consistent with the work of Nandi et al. 2011 [33] who described as goat were more sensitive to the infection than sheep which was characterized with a proliferative lesions in the first 1–2 months. Sex of the animal showed no association with prevalence of the disease and this might be due to the reason that both sex groups in the area were kept under the same management system.

Body condition of the animal was strongly correlated with prevalence of the virus in which poor body conditioned animals showed a higher prevalence comparing to good and medium body conditioned animals. This is associated with the development of strong immunity in well-nourished animals comparing to less nourished animals. The immune system protects animal health and contributes to animal well-being. Nutrition is an important modulator of immune function and can often balance between health and disease [34, 35]. The diet provides substrates such as energy and amino acids, that contribute to the development, maintenance and use of the immune system. In young animals, a severe deficiency of virtually any nutrient impairs many indices of immunocompetence [36]. Age of the study animals was also considered as a risk factor of infection but there was no significant difference among young and old animals although there was a slight difference in the prevalence of the disease and this might be because all animals in the study area were kept under similar husbandry and management systems. Regardless of disease prevalence, the severity of infection was higher in the young age group (< 1 year) due to the exposure to primary infections and poor immunity against infections. Nursing animals showed higher prevalence than non-nursing animals due to the difference in the chance of contact with the infected neonates during suckling period and in addition, nursing animals had more likely to develop stress and compromised immunity due to suckling which makes them more vulnerable for infection.

Management system of the animals were also another risk factor and animals kept under extensive management system and free housing developed more chance of infection due to high chance of contact and transmission of the virus. The effect of vaccination on developing infection was also explored and revealed that vaccinated animals were less likely to develop infection than non-vaccinated animals. The host immune response of Orf virus after vaccination has widely studied and many live attenuated vaccines can induce a strong immune response in vaccinated animals by stimulating lymph node originated lymphocytes to combat infections [37]. In addition, animals with treatment history of anthelmintic and antibiotics has also shown a less prevalence of viral infections and this is justified from the perspective of indirect effect on the inhibition of bacterial and parasitic infections which might help improving host immunity. Finally, study area also showed no difference in the incidence of the disease and epidemiologically, this finding might be interpreted from the point of having a similar agroecology for the survival of the virus and the management system of the animals and equal chance of exposure to infection in general.

## Conclusion

In conclusion, diagnosis of Orf based on traditional method is not accurate to identify the level of infection and as a result, we established a molecular diagnostic approach using PCR for confirmation of the virus and discriminating from other bacterial and viral pathogens. Future works will be focused on the detail phylogenetic analysis of the viral strains in all regions of the country.
